# Industry-specific prevalence and gender disparity of common mental health problems in the UK: A national repetitive cross-sectional study

**DOI:** 10.3389/fpubh.2023.1054964

**Published:** 2023-02-09

**Authors:** Shanquan Chen, Yuqi Wang

**Affiliations:** ^1^Department of Psychiatry, University of Cambridge, Cambridge, United Kingdom; ^2^Department of Computer Science, University College London, London, United Kingdom

**Keywords:** gender disparity, industrial classification, UK, workplace, common mental health problems

## Abstract

**Aims:**

The aim of the study was to evaluate the prevalence and temporal trend of common mental health problems (CMHPs) in the UK by industrial classification from 2012–2014 to 2016–2018 while evaluating the corresponding gender disparities.

**Methods:**

We used data from the Health Survey for England. CMPH was judged by a 12-item General Health Questionnaire. Industrial classifications were defined using the UK Standard Industrial Classification of Economic Activities. Data were fitted by the logistic models.

**Results:**

In this study, 19,581 participants covering 20 industries were included. In total, 18.8% of participants screened positive for CMHP in 2016–2018, which significantly increased from 16.0% in 2012–2014 [adjusted OR (AOR) = 1.17, 95% CI 1.08–1.27]. In 2016–2018, the prevalence of CMHP ranged from 6.2% in the industry of mining and quarrying to 23.8% in the industry of accommodation and food service activities. From 2012–2014 to 2016–2018, none of the 20 industries studied experienced significant decreases in the above prevalence; conversely, three industries saw significant increases, including wholesale and retail trade, repair of motor vehicles and motorcycles (AOR for trend = 1.32, 95% CI 1.04–1.67), construction (AOR for trend = 1.66, 95% CI 1.23–2.24), and other service activities, which cannot be classified (AOR for trend = 1.94, 95% CI 1.06–3.55). In total, 11 of the 20 industries studied had significant gender disparities against women, with the smallest gap being in the industry of transport and storage (AOR = 1.47, 95% CI 1.09–2.0) and the highest in the industry of arts, entertainment, and recreation (AOR = 6.19, 95% CI 2.94–13.03). From 2012–2014 to 2016–2018, gender disparities were narrowed only in two industries, including human health and social work activities (AOR for trend = 0.45, 95% CI 0.27–0.74), and transport and storage (AOR for trend = 0.5, 95% CI 0.27–0.91).

**Conclusion:**

The prevalence of CMHPs has increased and had a wide variation across industries in the UK. There were disparities against women, and the gender disparities have been keeping almost no improvement from 2012–2014 to 2016–2018.

## Background

Common mental health problems, such as depression and anxiety disorders, influence a wide population. In the UK, ~1 in seven people in the workplace experience mental health problems, and women are nearly two times as likely to have mental health problems as men (1). It was estimated that economic losses caused by mental health problems account for about 4.1% of the UK GDP (1), and better mental health support in the workplace can save UK businesses up to £8 billion per year (2). Reforms in the UK over the past decade resulted in advancing achievement in the integration of employment and mental health, as well as considerable related outcomes such as helping employed people move off sickness absence (3). However, a recent official report indicated that still more than 50% of all sickness absence days can be attributed to mental health conditions (4).

People are exposed to their unique occupational environments, which depend on the type of industry (such as construction) they belong to. A cross-sectional survey based on 40,986 police employees in the UK indicated that 9.8% of the participants had probable depression, with 12.45% of women vs. 8.24% of men (5). A cross-sectional survey based on 78 emergency ambulance service workers in the UK indicated that 53.8% of respondents experienced work-related burnout and that those most at risk of burnout were full-time working men (6). A cross-sectional survey of 5,497 workers found that clerical and secretarial, sales, and personal and protective services were occupations that usually had a higher prevalence of common mental health problems (CMHPs), whereas craft and related, and plant and machine operatives had a lower prevalence of CMHPs, compared to the overall prevalence in all adults (7). Understanding mental health by industrial classification could be conducive to the individualization and pertinence of policies or intervention measures, especially given that rules and regulations are usually formulated according to the industry. However, based on our knowledge, no study in the UK looked at mental health problems from the perspective of industrial classification.

This study aimed to evaluate the prevalence and temporal trend of CMHPs in the UK by industrial classification from 2012–2014 to 2016–2018 while evaluating the corresponding gender disparities.

## Methods

### Database and participants

We used data from the Health Survey for England (HSE). HSE is a representative repeated cross-sectional survey of people aged 13 or over in England, looking at changes in the health and lifestyles of people all over the country. It has been widely used by central and local governments in the UK for decision-making (8). The HSE uses stratified multistage probability sampling to select samples. In the first stage, a random sample of primary sampling units (PSUs), based on postcode sectors, was selected. Within each selected PSU, a random sample of postal addresses (known as delivery points) was then drawn (9). This design ensures that every address in England has an equal chance of being included in the survey each year, and the results are representative of the population living in private households. Approximately 8,000 adults and 2,000 children take part in the HSE each year. Information is collected through an interview and, if participants agree, a visit from a specially trained nurse. Collected items included socio-economic and demographic characteristics and validated measures of mental disorder symptoms (discussed below). Detailed descriptions of HSE, such as sampling methods and quality-control procedures, can be found elsewhere (9).

This study used the survey waves of 2012, 2014, 2016, and 2018, because the industrial classification has been kept as the same version since 2012, and data on CMHPs are collected every 2 years (discussed below). To increase the available sample size to improve the precision of our estimates, we combined 2012 and 2014 as the starting period (named 2012–2014), as well as 2016 and 2018 as the ending period (named 2016–2018).

**Figure 1:**
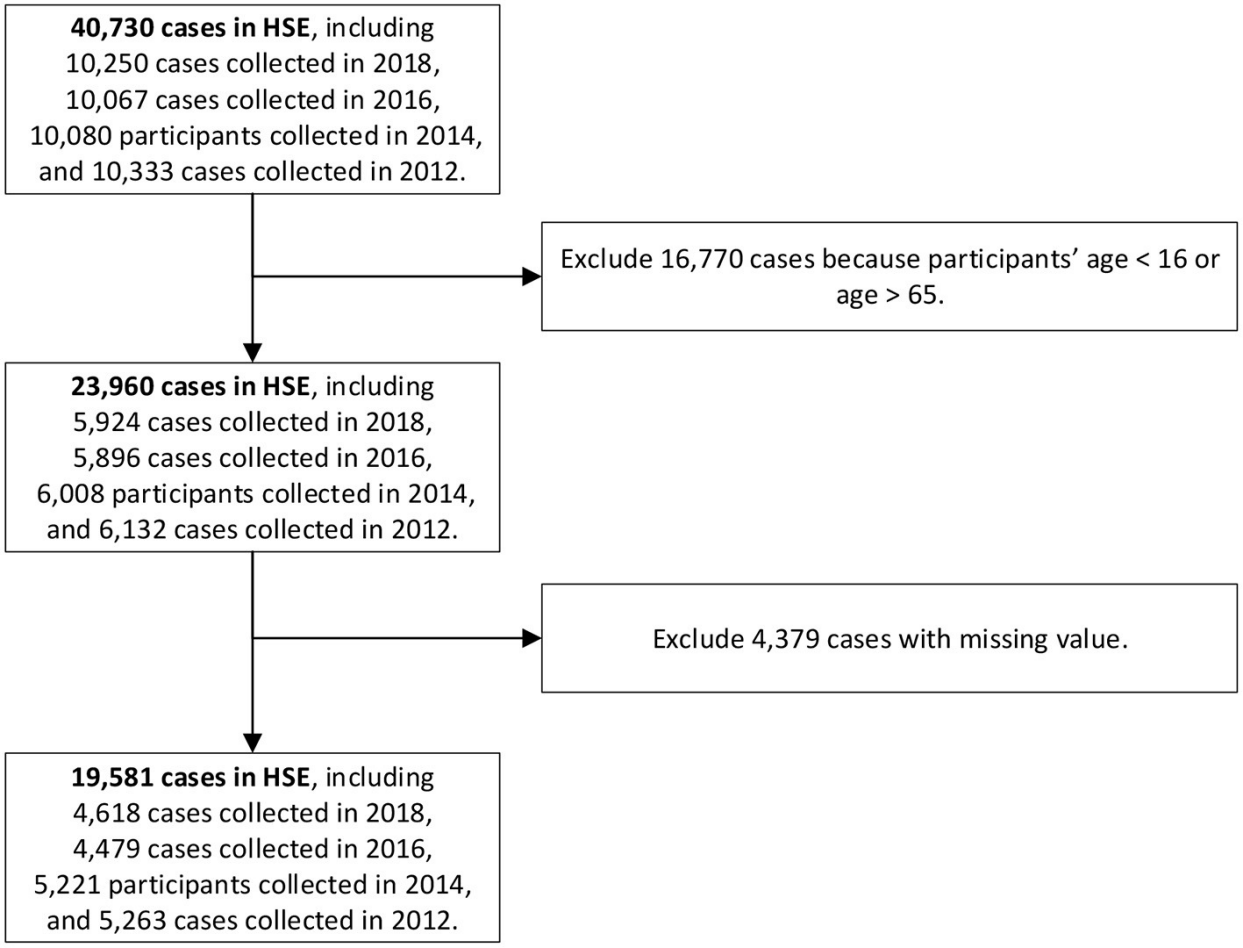
Inclusion and exclusion of the participants. HSE, Health Survey for England.

In this study, we only included those aged between 16 and 65, considering the legal working age and retirement age (66 years) in the UK. We excluded the cases with missing values [4,379 (18.3%) records] instead of any imputation. Figure 1 shows the selection of the cases in detail.

This study was carried out in accordance with the Declaration of Helsinki. The data are publicly available. The use of secondary de-identified data made this study exempt from institutional review board review. Participants in the original studies gave informed consent and each study was approved by the London Medical Research Ethics Council and/or Local Research Ethics Councils before each annual data collection cycle (8, 9).

### Measures

#### Common mental health problems

Common mental health problems were judged by the 12-item General Health Questionnaire (GHQ-12). This instrument concentrates on the broader symptoms of psychological morbidity and measures the characteristics such as general levels of happiness, depression, anxiety, sleep disturbance, and self-confidence. Each of the 12 items was rated on a four-point response scale to indicate whether symptoms of mental ill health were “not at all present,” “no more than usual,” “rather more than usual,” or “much more than usual,” with first two coded as 0 and last two coded as 1. Then, GHQ-12 is scored on a range from 0 to 12, and a validated score of 4 or more is indicative of probable mental health problems with a sensitivity of 0.69 and specificity of 0.88 (8, 10, 11).

#### Industrial classification

Industrial classifications were defined using the level 1 of the UK Standard Industrial Classification of Economic Activities (UK-SIC, version 2007), including accommodation and food service activities; administrative and support service activities; agriculture, forestry, and fishing; arts, entertainment, and recreation; Construction; education; electricity, gas, steam, and air conditioning supply; financial and insurance activities; human health and social work activities; information and communication; manufacturing; mining and quarrying; professional, scientific, and technical activities; public administration and defense; compulsory social security; real estate activities; transport and storage; water supply, sewerage, waste management and remediation activities; wholesale and retail trade; repair of motor vehicles and motorcycles; and other service activities (12). The original industrial classifications did not cover non-employed workers. Considering the mutual transformation between employed and non-employed workers, and to broaden the scope of this study, in this study, we included the non-employed as a separate group in the industrial classification.

#### Other variables

We investigated the following socio-demographic characteristics, including age (16–24, 25–34, 35–44, 45–54, and 55–65), gender (male vs. female), marital status (married/cohabitation, never married, and widowed/divorced/separated), education attained (less than secondary education, secondary general or vocational education, tertiary education, and foreign/other qualifications), and socio-economic status [measure by the index of multiple deprivations, the official measure of relative deprivation in England (13)]. We also investigated long-standing physical illnesses (such as back or neck pain, and disabilities; categorized as yes or no) because of their evidenced influence on psychological health in the workplace (14).

### Statistical analysis

Data were analyzed for each industrial classification separately. This makes within-industrial comparisons (analyses of changes over time) robust to any possible between-industrial differences. Repeated cross-sectional sampling is a standard method for measuring changes (15, 16), including for the assessment of trends relating to depression based on the screening tools (17). Survey weighting was used to adjust for the complex survey design, including the clustering, and stratification, to make the estimates representative of each year. The weight values were provided directly in the HSE datasets. Details of how the weights were calculated can be found elsewhere (18, 19). The prevalence was calculated as the proportion of participants scoring 4+ on the GHQ-12.

To estimate the temporal trend, we fitted industrial-specific weighted logistic regression models, with CMHPs (yes or no) as the dependent variable and period (a binary variable with 2012–2014 coded as 0 and 2016–2018 coded as 1) as the key predictor, controlling for age, gender, marital status, education attained, socio-economic status, and long-standing physical illness (Equation 1).


(1) $$\begin{array}{l} CMHP=\alpha +\;period+confunders+\varepsilon \end{array}$$

To estimate the gender disparities, we fitted industrial-specific weighted logistic regression models, with CMHPs (yes or no) as the dependent variable and gender (a binary variable with male coded as 0 and female coded as 1) as the key predictor, controlling for age, marital status, education attained, socio-economic status, long-standing physical illness, and study period (Equation 2). To explore how gender disparities had changed, we added an interaction term between gender and period (Equation 3).


(2) $$\begin{array}{l} CMHP=\alpha +\;gender+confunders+\varepsilon \end{array}$$

(3) $$\begin{array}{r} CMHP=\alpha +\;gender+\;period+gender\times period+\\ \;confunders+\varepsilon \; \end{array}$$

All analyses were conducted in R version 3.6.0 (20). A *p*-value of < 0.05 was considered statistically significant. The results are reported following the STROBE checklist for cohort studies.

## Results

In this study, 19,581 out of 40,730 participants, aged between 16 and 65, were included (9,097 participants in the start phase, 2012–2014, and 10,484 participants in the end phase, 2016–2018; Figure 1). Table 1 summarizes their basic characteristics. Among these participants, 56.6% were female and 66.5% were married or in cohabitation. People aged 45–55 accounted for 24.2%, followed by people aged 35–44 (22.6%). In total, 12.8% of participants were from the industry of human health and social work activities, followed by manufacturing (12.0%).

**Table 1:** Basic description.

**Characteristic**	**No. (%) of participants**
**Gender (=female)**	11,079 (56.6%)
**Age**	
16–24	2,355 (12.0%)
25–34	3,771 (19.3%)
35–44	4,428 (22.6%)
45–54	4,730 (24.2%)
55–65	4,297 (21.9%)
**Education attained**	
Less than secondary education	2,366 (12.1%)
Secondary general or vocational education	10,828 (55.3%)
Tertiary education	6,315 (32.3%)
Foreign/other qualifications	72 (0.4%)
**Marital status**	
Married/cohabitation	13,022 (66.5%)
Never married	4,580 (23.4%)
Widowed/divorced/separated	1,979 (10.1%)
**Social economics status**	
Most deprived	3,812 (19.5%)
2	3,562 (18.2%)
3	3,802 (19.4%)
4	4,215 (21.5%)
Lest deprived	4,190 (21.4%)
**Long-lasting illness (=yes)**	7,032 (35.9%)
**Study period**	
2012–2014	9,097 (46.5%)
2016–2018	10,484 (53.5%)
**Industrial classifications**	
Accommodation and food service activities	869 (4.4%)
Administrative and support service activities	1,059 (5.4%)
Agriculture, forestry, and fishing	207 (1.1%)
Arts, entertainment, and recreation	335 (1.7%)
Construction	1,622 (8.3%)
Education	1,783 (9.1%)
Electricity, gas, steam, and air conditioning supply	151 (0.8%)
Financial and insurance activities	756 (3.9%)
Human health and social work activities	2,497 (12.8%)
Information and communication	875 (4.5%)
Manufacturing	2,356 (12%)
Mining and quarrying	83 (0.4%)
Non-employed	467 (2.4%)
Other service activities	340 (1.7%)
Professional, scientific and technical activities	1,361 (7.0%)
Public administration and defense; compulsory social security	1,088 (5.6%)
Real estate activities	155 (0.8%)
Transport and storage	1,273 (6.5%)
Water supply, sewerage, waste management and remediation activities	143 (0.7%)
Wholesale and retail trade; repair of motor vehicles and motorcycles	2,161 (11.0%)

Data are shown as numbers (percentages).

Among included participants, 18.8% (95% CI 18.0–19.6) of them screened positive for CMHPs in 2016–2018, which significantly increased from 16.0% (95% CI 15.2–16.8) in 2012–2014 [adjusted OR (AOR) = 1.17, 95% CI 1.08–1.27].

**Figure 2:**
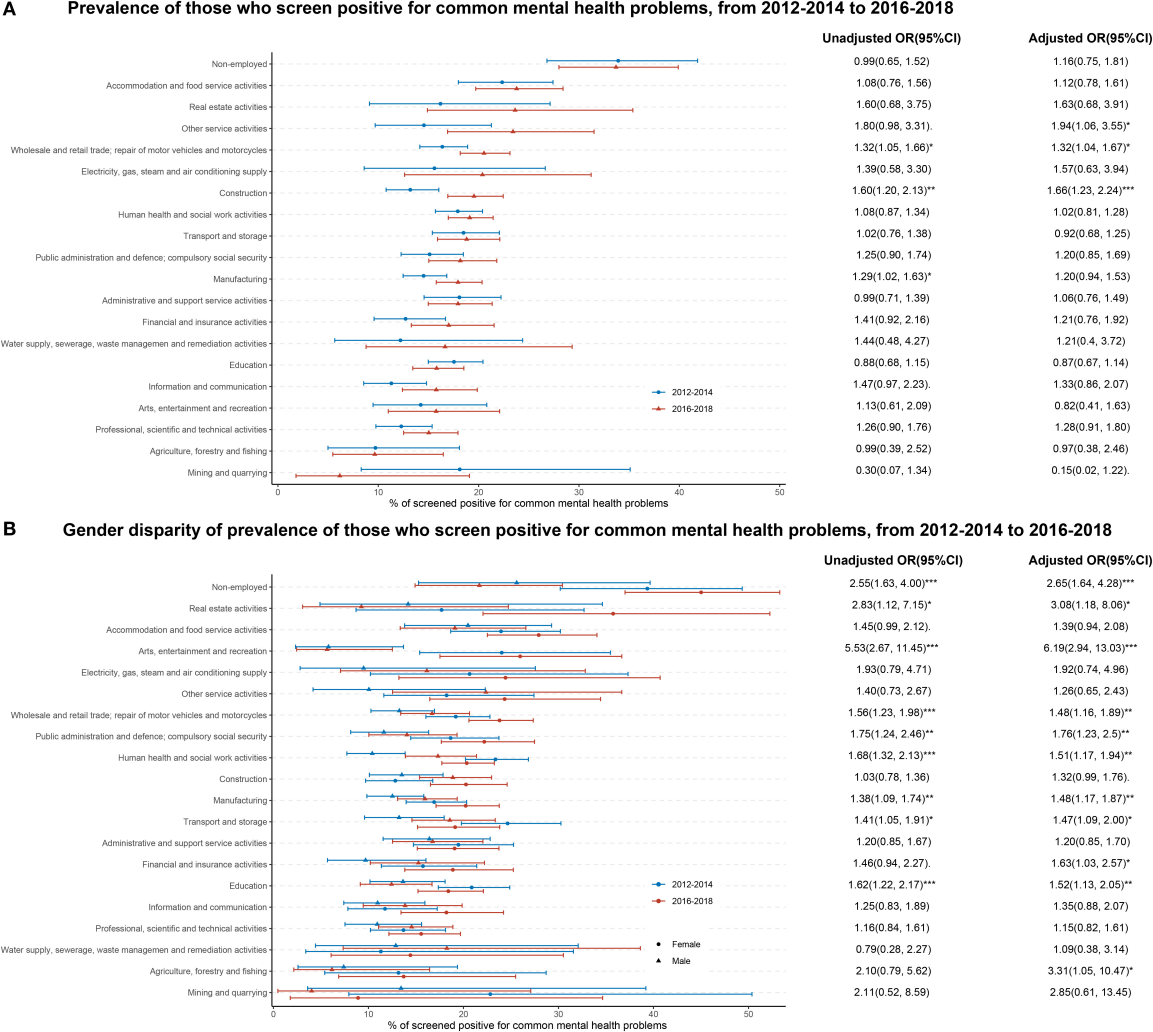
Prevalence and gender dispraise of those who had common mental health problems in the UK, by industrial classification, from 2012–2014 to 2016–2018. Shown are the prevalence and its 95% confidence interval (CI) of populations who screen positive for common mental health problems. The odd ratios (OR) and its 95 CI% were estimated from weighted logistic with depression (yes or no) as the dependent variable and survey period [with 2012–2014 as the reference, in **(A)**] or gender [with male as the reference, in **(B)**] as the key predictor. Adjusted OR controlled for age, gender **(A)** or study period **(B)**, marital status, education attained, index of multiple deprivations, and long-standing physical illness. ^*^ < 0.05, ^**^ < 0.01, ^***^ < 0.001.

Large variations in the prevalence of CMHPs were observed across industries (Figure 2A). In 2016–2018, this prevalence ranged from 6.2% (95% CI 1.8–19.1) in the industry of mining and quarrying, to 23.8% (95% CI 19.7–28.4) in the industry of accommodation and food service activities, and to 33.7% (95% CI 28.0–39.9) in the non-employed (Figure 2A). Figure 2A also indicated that from 2012–2014 to 2016–2018, none of the industries experienced significant decreases in the prevalence; on the contrary, some industries had significant increases, including the industry of wholesale and retail trade, repair of motor vehicles and motorcycles (AOR = 1.32, 95% CI 1.04–1.67), the industry of construction (AOR = 1.66, 95% CI 1.23–2.24), and the industry of other service activities, which cannot be classified (AOR = 1.94, 95% CI 1.06–3.55).

Gender disparities in the prevalence of CMHP also varied widely across industries. In total, 11 of the 20 industries studied had significant gender disparities against women, with the smallest gap being in the industry of transport and storage (AOR = 1.47, 95% CI 1.09–2.0) and the highest in the industry of arts, entertainment, and recreation (AOR = 6.19, 95% CI 2.94–13.03; Figure 2B). From 2012–2014 to 2016–2018, gender disparities were narrowed only in two of the 11 industries above, including the industry of human health and social work activities (AOR for trend = 0.45, 95% CI 0.27–0.74) and the industry of transport and storage (AOR for trend = 0.5, 95% CI 0.27–0.91; Table 2).

**Table 2:** Temporal trend of gender disparity of prevalence of those who screen positive for common mental health problems, from 2012–2014 to 2016–2018.

**Industrial classification**	**OR for trend** **(95% CI)^a^**
Non-employed	1.61 (0.62, 4.21)
Real estate activities	4.05 (0.58, 28.24)
Accommodation and food service activities	1.32 (0.61, 2.86)
Arts, entertainment and recreation	1.19 (0.27, 5.31)
Electricity, gas, steam, and air conditioning supply	0.77 (0.11, 5.30)
Other service activities	0.50 (0.12, 2.05)
Wholesale and retail trade; repair of motor vehicles and motorcycles	1.07 (0.66, 1.74)
Public administration and defense; compulsory social security	1.00 (0.50, 2.00)
Human health and social work activities	0.45 (0.27, 0.74)^**^
Construction	1.27 (0.71, 2.28)
Manufacturing	0.92 (0.57, 1.48)
Transport and storage	0.50 (0.27, 0.91)^*^
Administrative and support service activities	0.92 (0.46, 1.83)
Financial and insurance activities	0.91 (0.36, 2.30)
Education	0.99 (0.55, 1.76)
Information and communication	1.28 (0.55, 2.96)
Professional, scientific, and technical	0.85 (0.43, 1.67)
Water supply, sewerage, waste management, and remediation activities	1.17 (0.12, 11.17)
Agriculture, forestry, and fishing	1.36 (0.19, 10.02)
Mining and quarrying	2.71 (0.02, 296.71)

a The odd ratios (ORs) for trend and its 95% CI were estimated by adding an interaction term between gender and study period into the adjusted logistical model in Figure 2B.

* < 0.05,

** < 0.01, ^***^ < 0.001.

## Discussion

### Statement of principal findings

The prevalence of those who were screened positive for CMHPs increased from 2012–2014 to 2016–2018. None of the 20 industries that were studied experienced decreases in this prevalence, but three industries had significant increases. Salient gender disparities against women were detected in 11 of the 20 industries studied, and among these 11 industries, the gender disparities were narrowed only in two industries from 2012–2014 to 2016–2018.

### Possible explanations and comparisons with other studies

The consistently highest prevalence and the non-improvement situation among the non-employed from 2012–2014 to 2016–2018 deserve our attention, although we cannot ignore UK's achievement in helping these people back to work. Previous studies have evidenced the mutual exacerbation of non-employment and common mental health problems and also indicated that the welfare system in the UK does not seem to nullify the effect of non-employment on mental health (21, 22). Besides the existing non-employment benefit and back-to-work support, public policy should therefore also focus on the early prevention of mental health problems among the non-employed. In addition, gender disparities with a higher prevalence in women also emphasize that more attention should be paid to female non-employed.

Our findings highlighted some high-risk industries for CMHP and revealed the blind spots in existing mental health studies, including industries of accommodation and food service activities; real estate activities; wholesale and retail trade, repair of motor vehicles and motorcycles; and other services activities, which cannot be classified. These industries usually involve working face-to-face with the general public, involve a degree of responsibility coupled with some unpredictability in how their clients might behave toward them, or involve irregular and long working hours (23). Thus, working in these industries is emotionally demanding and exposes the employees to adverse social behavior (such as violence and verbal aggression), contributing to the important sources of psychological risks in the workplace (7). However, attention from the previous literature to these industries is insufficient, when compared to industries whose voices, in terms of psychological demands, are more likely to be heard due to their high physical-risk occupational environment (such as mining and quarrying, agriculture, forestry and fishing, and construction) or the fact that they are the industries where researchers primary come from (such as professional, scientific and technical activities, and human health and social work activities).

This study also highlighted the insufficient attention to the against-females-disparities on the prevalence of CMHPs in the workplace, as evidenced by the fact that gender gaps in most (nine in 11) of the industries have not been narrowed from 2012–2014 to 2016–2018. Although gender disparities in the industry of human health and social work activities and the industry of transport and storage were narrowed, this improvement was to some extent unreasonable as this narrowing resulted from men being worse off (increased prevalence) and women being better off (decreased prevalence). Previous studies have identified some risk factors, which have gender-specific impacts on mental health (24, 25). For instance, working full-time decreases the risk of mental problems among men, but not among women; fixed-term contract only increases the risk of mental problems among women; men are more affected by the changes in tasks and a lack of pride at work, while mental problem drivers in women are no training, low motivation, and weak social support at work; worrying of involuntary interruptions during work is also disproportionally affect women (24, 25). However, existing evidence cannot explain our findings that the against-females-disparities in the prevalence of CMHPs were only identified in part of (11 of the 20) industries studied not all. Both industry- and gender-specific factors need to be identified in future studies. In addition, our findings that the against-females-disparities in female-dominated areas, such as the industry of education, to some extent contradict the evidence from Denmark, where mental disorders were higher for men working in female-dominated areas (26). These findings may imply the possible influence of cultural differences. The underlying reasons should be explored in future studies and call for more corresponding interventions.

### Strengths and limitations

To the best of our knowledge, we first evaluated the prevalence of those who had CMHPs by industrial classification in the UK. The repetitive cross-sectional representative data enabled the exploration of the temporal trend of this prevalence. The gender disparity we explored allowed a more nuanced and practical assessment of previous achievements. This study identified the industries where prevalence and gender disparities were relatively higher and where we should focus in the future.

Our study was limited by the use of self-reported data, which may be subjected to recall bias. Second, the outcome measured by GHQ-12 is not equal to the clinical diagnosis. Third, the self-administered instruments have only been validated for binary detection of depressive disorders and do not provide accurate quantification of severity. Fourth, people with CMHPs may have been successfully treated and thus without residual symptoms to be identified by the survey instruments; such people would have been missed by this approach, underestimating the proportion of people who had common mental health problems.

One unanswered question of this study is that we identified the industries where prevalence and gender disparities were relatively higher and where we should focus in the future, but we were unable to further explore what are the contributing factors, primarily due to the lacking of industry-specific variables, such as the irregular working hours and adverse social behavior we mentioned (7, 23). More studies are needed to explore mental health from the perspective of the industry. In addition, more specific surveys or data with industry-specific information are also needed.

## Conclusion

The prevalence of CMHPs has increased and had a wide variation across industries in the UK. There were disparities against women, and the gender disparities have been keeping almost no improvement from 2012-−2014 to 2016–2018. People are exposed to their unique occupational environments, which depend on the type of industry they belong to. Our findings can be used by the pertinence of policies or intervention measures by industries, given that rules and regulations are usually formulated according to the industry.

## Data availability statement

Publicly available datasets were analyzed in this study. This data can be found at: https://beta.ukdataservice.ac.uk/datacatalogue/series/series?id=2000021.

## Ethics statement

The studies involving human participants were reviewed and approved by the London Medical Research Ethics Council and/or Local Research Ethics Councils prior to each annual data collection cycle. The patients/participants provided their written informed consent to participate in this study.

## Author contributions

SC had full access to all of the data in the study, takes responsibility for the integrity of the data and the accuracy of the data analysis, acquisition, analysis, interpretation of data, statistical analysis, administrative, technical, material support, and supervision. SC and YW: concept, design, drafting of the manuscript, and critical revision of the manuscript for important intellectual content. All authors contributed to the article and approved the submitted version.
